# Supplementary material for: Amplifying and Fine-Tuning Rsm sRNAs Expression and Stability to Optimize the Survival of Pseudomonas brassicacerum in Nutrient-Poor Environments
Source: Microorganisms. 2021 Jan 26;9(2):250. doi: 10.3390/microorganisms9020250 (PMC7911923; doi:10.3390/microorganisms9020250)
Supplement: Supplementary file 1 [file microorganisms-09-00250-s001.pdf]

Table S1. Primers, plasmids and strains used in this study

Primers used in this study

Gene	Primer	Description
		Transcriptional *lacZ* fusions
rsmX-2	rsmX-2-lacZ-EcoRI	CTTGAATTCGGAGTCGTAATGGTCTTGG, located upstream of *rsmX-2* (-144 to -125)
	rsmX-2-lacZ-PstI	CTTCTGCAGCCCTGTACCCAGTGGATG, located in *rsmX-2* gene (+2 to +19)
rsmY	rsmY-lacZ-EcoRI	CATAGAATTCAATCATGACGATGTGTAAGCATTATC, located upstream of rsmY (-89 to -64)
	rsmY-lacZ-PstI	AAACTGCAGGCGCTACATCCATGGAAGC, located in rsmY gene (-6 to +13)
		Overexpressions
rsmX-2	rsmX-2-PstI	GTTCTGCAGCCACTGGGTACAGGGAG, forward primer of *rsmX-2*
	rsmX-2-KpnI	CTTGGTACCCAGGCTACTTTTTCACAGACG, reverse primer of *rsmX-2*
		5' RACE
	DT88	GAAGAGAAGGTGGAAATGGCGTTTTGG
	DT89	CCAAAACGCCATTTCCACCTTCTCTTC
rsmX-2	rsmX-2-R1	GAAAAAAAACCCGCCTAGGC, reverse primer in *rsmX-2* (+91 to + 110)
	rsmX-2-R2	CCTAGGCGGGTTTTTTGAGG, reverse primer in *rsmX-2* (+78 to + 98)

Plasmids used in this study

Plasmid	Description	Reference
	Transcriptional *lacZ* fusions	
pME6016	pVS1-p15A shuttle vector for transcriptional *lacZ* fusions, Tc^r	Schnider-Keel *et al.*, 2000
pME6016-*rsmX-2-lacZ*	pME6016 derivate containing a transcriptional *rsmX-2*'-'lacZ fusion; Tcr	This study
pME6016-*rsmX-lacZ*	pME6016 derivate containing a transcriptional *rsmX'*-'*lacZ* fusion; Tc^r	Lalaouna *et al.*, 2011

pME6016-*rsmY-lacZ*	pME6016 derivate containing a transcriptional *rsmY'*-'*lacZ* fusion; Tc^r	Lalaouna *et al.*, 2011
pME6016-*rsmZ-lacZ*	pME6016 derivate containing a transcriptional *rsmZ'*-'*lacZ* fusion; Tc^r	Lalaouna *et al.*, 2011
	Overexpressions	
pME6032	*Nru*I-*Eco*RI $lacI^q$-P_{tac} fragment of pJF118EH subcloned in [*Bam*HI]-*Eco*RI-digested pME6031; $lacI^q$-P_{tac} expression vector	Heeb *et al*., 2002
pME6032-*gacA*	pME6032 derivate containing *gacA* gene	Lalaouna *et al.*, 2011
pME6032-*rsmX*	pME6032 derivate containing *rsmX* gene	Lalaouna *et al.*, 2011
pME6032-*rsmX-2*	pME6032 derivate containing *rsmX-2* gene	This study
pME6032-*rsmY*	pME6032 derivate containing *rsmY* gene	Lalaouna *et al.*, 2011
pME6032-*rsmZ*	pME6032 derivate containing *rsmZ* gene	Lalaouna *et al.*, 2011

Strains used in this study

Strain	Description	Reference
	Escherichia coli	
TOP10	F- mcrA Δ(mrr-hsdRMS-mcrBC) ϕ80lacZΔM15 ΔlacX74 nupG recA1 araD139 Δ(ara-leu)7697 galE15 galK16 rpsL(Strr) endA1 λ-	Laboratory strain
GM2163	F- dam-13::Tn 9 dcm-6 hsdR2 leuB6 his-4 thi-1 ara-14 lacY1 galK2 galT22 xyl-5 mtl-1 rpsL136 tonA31 tsx-78 supE44 McrA- McrB	Laboratory strain
S17-1	thi thr leu tonA lacY supE recA::RP4-2-Tc::Mu, Kn::Tn7	Laboratory strain
	Pseudomonas brassicacearum	
Phase I NFM421	Wild type	Achouak *et al*., 2000
NFM421 Δ*gacA*	Δ*gacA*	Lalaouna *et al.*, 2011

TaqMan

Gene	Primer/probe	Description
Primers		
16S	16S-F	CGGAATTACTGGGCGTAAAGC
	16S-R	CAGTGTCAGTATCAGTCCAGG
rsmX-1	rsmX-1-F	GTTCTGCAGTCCACTGAAGCACAGGAAGT
	rsmX-1-R	GACCATTACGACTCCCTGTC
rsmX-2	rsmX-2-F	CATCCACTGGGTACAG*GG*
	rsmX-2-R	CCTAGGCGGGTTTTTTGAGG
rsmY	RsmY-F	CATGGATGTAGCGCAGGACG
	RsmY-R	CCGCCGAAGCGGGGCTTTGC
rsmZ	rsmZ-F	TGTCGACGGACAGACACAC
	rsmZ-R	CCTAATCCCTTTAATCCTTTTCATC
Probes		
16S	TaqMan-16S	CTCAACCTGGGAACTGCATTCAAAACTGTC
rsmX-1	TaqMan-rsmX-1	CAGGATCAGGGACGATCGACCTTGC
rsmX-2	TaqMan-rsmX-2	CATGGAATGCCGGACAGGGAGTC
rsmY	TaqMan-rsmY	CGGCAGAGTGAAATGGATGTCAGGG
rsmZ	TaqMan-rsmZ	CGTCAAGGACGATGGGAAGGAAGGACATCGCAGG

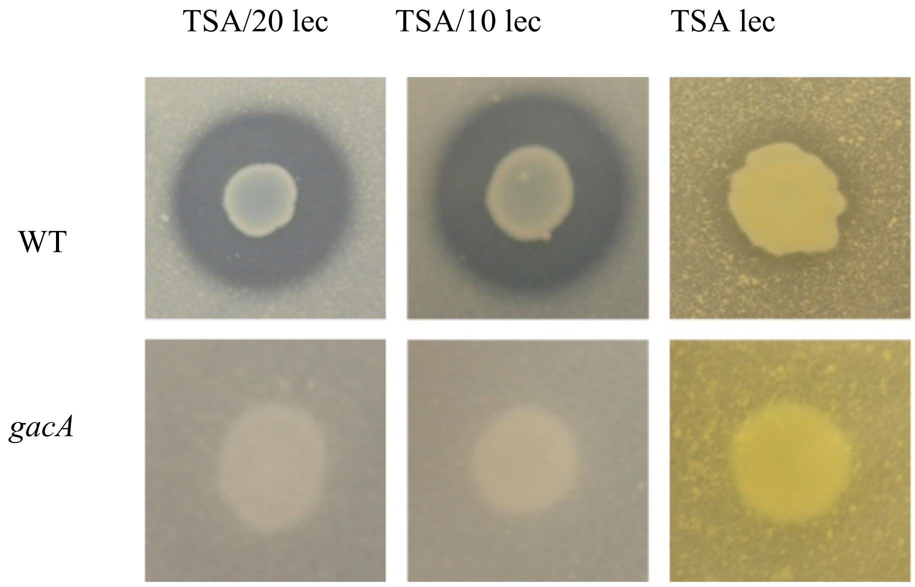

Strains	Media		
	TSA/20	TSA/10	TSA
WT	0.65±0.005	0.62±0.03	0.15±0.05
gacA	0	0	0

Figure S1. Detection of protease activity under nutrient starvation conditions. (A) Wild-type and *gacA* strains on TSA/20 lec, on TSA 1/10 lec and on undiluted TSA lec media. (B) Halo thickness (distance from bacteria to halo edge) is expressed as the mean of three measurements, in cm.

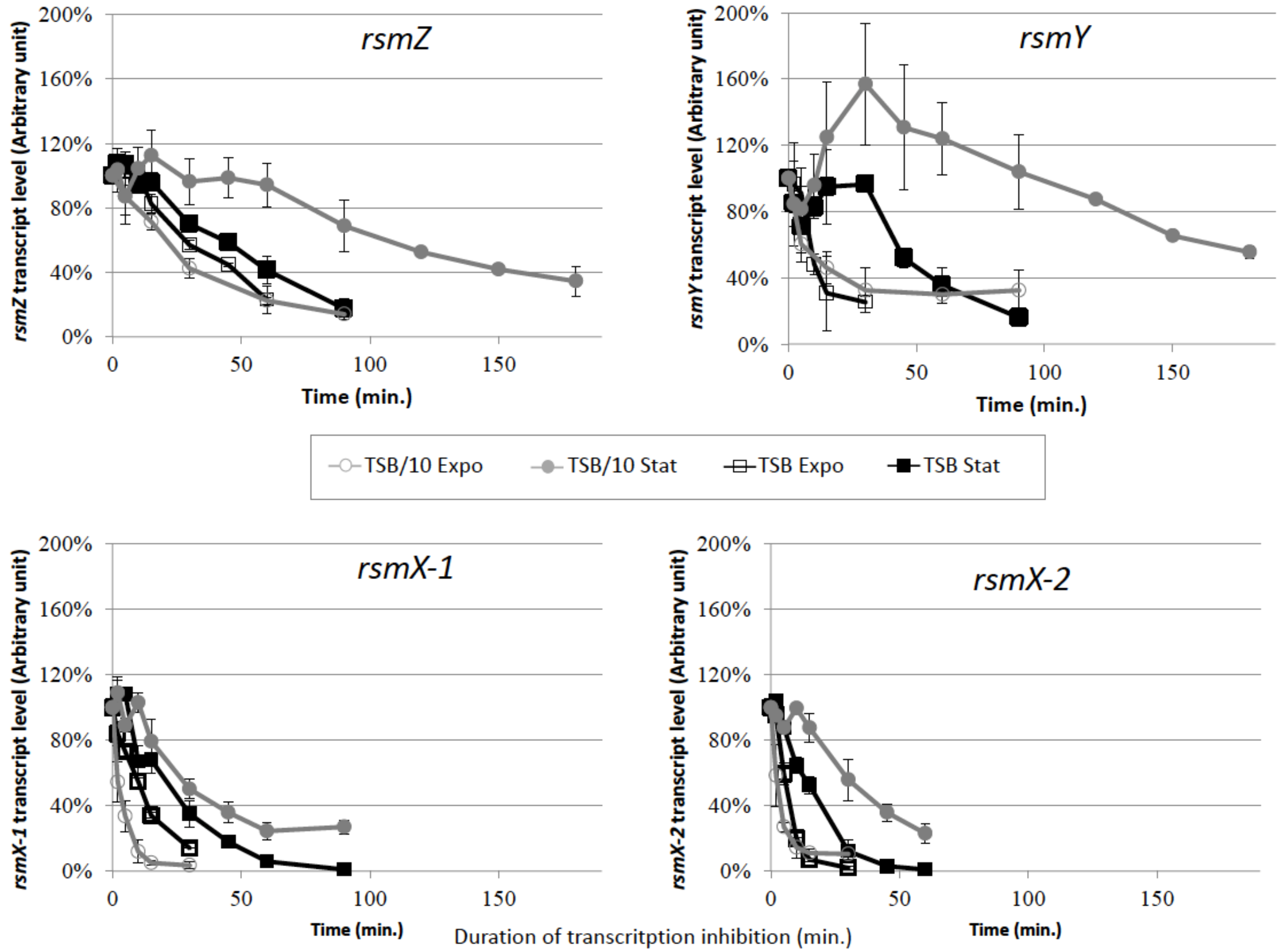

Figure S2: Half-lives of RsmX-1, RsmX-2, RsmY and RsmZ of *P. brassicacearum* NFM421.
